# VEGF receptors on PC12 cells mediate transient activation of ERK1/2 and Akt: comparison of nerve growth factor and vascular endothelial growth factor

**DOI:** 10.1186/1477-5751-5-8

**Published:** 2006-06-01

**Authors:** Ingrid Berger, Sonja Stahl, Natalia Rychkova, Ute Felbor

**Affiliations:** 1 Department of Human Genetics, University of Würzburg, Germany

## Abstract

Vascular endothelial growth factor (VEGF) and endostatin are angiogenic and anti-angiogenic molecules, respectively, that have been implicated in neurogenesis and neuronal survival. Using alkaline phosphatase fusion proteins, we show that the PC12 neuronal cell line contains cell membrane receptors for VEGF but not for endostatin and the collagen XV endostatin homologue. Immunocytochemistry confirmed that proliferating and differentiated PC12 cells express VEGF receptors 1, 2 and neuropilin-1. While no functional effects of VEGF on PC12 cell proliferation and differentiation could be observed, a slight VEGF-induced reduction of caspase-3 activity in differentiated apoptotic PC12 cells was paralleled by transient activation of ERK1/2 and Akt. In direct comparison, nerve growth factor proved to be a strikingly more potent neuroprotective agent than VEGF.

## Background

VEGF, VEGF receptor antagonists, and the C-terminal collagen XVIII fragment endostatin, an inhibitor of angiogenesis and tumor growth [1], have been tested for use in long-term therapies to enhance or reduce vascularisation [2]. Therefore, knowledge of VEGF and endostatin receptor expression patterns as well as of their non-endothelial cell functions is important. VEGF was originally identified as a vascular permeability factor [3] which turned out to be crucial for vasculo- and angiogenesis [4]. Later, non-endothelial VEGF target cells have been described in a variety of organs [5]. More recently, autocrine and paracrine functions have been observed in neurogenesis and neuronal survival in vitro and in vivo, both in the central nervous system and the peripheral nervous system [6]. Endostatin was implicated in neuronal cell migration and axon guidance in *Caenorhabditis elegans *[7]. Fc-endostatin dimers were also reported to have motogenic activity on rat pheochromocytoma PC12 cells cultured on Matrigel [8], an extracellular matrix preparation used for differentiation of endothelial cells into tube-like structures. NGF-treated PC12 cells are an established model for analysis of neuronal differentiation, neuronal survival and neurotrophin signal transduction [9]. Finally, increased neuronal and paracellular endostatin deposits were found in patients with Alzheimer's disease [10].

VEGF exerts its anti-apoptotic effect on hypoxic neurons via VEGF receptor 2 (VEGFR-2), neuropilin-1 (NRP1), the Ras/mitogen-activated protein kinase (MAPK) and the phosphatidylinositol 3-kinase (PI3K)/Akt kinase pathways [11-13] as in VEGFR-2-dependent endothelial survival [14]. Ras/MAPK and PI3K/Akt are also involved in PC12 cell survival signaling stimulated by nerve growth factor (NGF) [15,16]. Since VEGF has also been suggested to act as a neurotrophin in motoneuron degeneration [17], we intended to evaluate the effects of VEGF and endostatins on neuronal differentiation and survival in direct comparison with the prototypic neurotrophin NGF. PC12 cells were first probed with dimeric fusion proteins composed of the human placental isozyme of alkaline phosphatase (AP) at the N-terminus and murine (m) VEGF_164 _or endostatins at the C-terminus. While the endostatin affinity probes did not react with PC12 cells, AP-mVEGF_164 _strongly bound to proliferating and differentiated PC12 cells. Although PC12 cells were subsequently shown to express VEGF receptors 1, 2 and neuropilin-1, only a minor neuroprotective effect was observed for VEGF when compared to NGF.

## Materials and methods

### Cell culture

PC12 cells were a gift from Drs. M. Sendtner and S. Wiese (Department of Neurology, University of Wuerzburg, Germany). Cow pulmonary artery endothelial (CPAE) cells were purchased from ATCC (CCL-209). PC12 cells were cultured in DMEM with glutamax-I (Gibco) supplemented with 10% horse serum, 5% fetal bovine serum, 100 U/ml penicillin G, and 100 μg/ml streptomycin (Gibco) in 5% CO_2 _at 37°C. For differentiation experiments, PC12 cells were plated on poly-L-ornithine coated tissue culture dishes and allowed to adhere over night (o/n). After one wash with serum-free DMEM, the cells were differentiated in serum-free DMEM containing 50 ng/ml human recombinant NGF (PAN Biotech) for 3 days [18]. Although Fc-endostatin dimer application induced the formation of multicellular PC12 aggregates on Matrigel [8], Matrigel was not chosen for the current study since it is an extracellular matrix preparation generally used for endothelial tube formation assays.

### Alkaline phosphatase staining of PC12 cells

For construction and expression of AP fusion proteins see [19]. PC12 cells were either grown to 80% confluence or differentiated in 6-well plates, and AP staining was performed as described in [20]. Staining was monitored with a Nikon Eclipse TE2000-U inverted microscope and documented using the Spot Insight QE Color imaging software (Visitron). Quantitative measurement of AP fusion protein binding to proliferating PC12 cells was carried out as described previously [21].

### Immunocytochemistry

PC12 cells plated on poly-L-ornithine coated glass coverslips were fixed in phosphate-buffered saline (PBS) containing 4% paraformaldehyde at room temperature for 20 min, washed three times in prewarmed tris-buffered saline (TBS) for 5 min, and incubated in blocking buffer (TBS with 10% goat serum) at room temperature for 1 h. After washing once with prewarmed TBS, the cells were incubated with anti-Flt-1 (sc-316), anti-Flk-1 (sc-504) or anti-Neuropilin (sc-5541) antibodies (2 μg/ml, Santa Cruz Biotechnology) in blocking buffer at 4°C o/n. Unbound primary antibodies were removed by washing, and the cells were incubated in 20 mM ammonium chloride solution for 30 min to reduce autofluorescence. Cells were stained for 1 h at 37°C with a secondary Cy3-conjugated goat anti-rabbit antibody (1:500, Dianova) in blocking buffer. After washing, the coverslips were mounted in Kaiser's glycerol gelatine (Merck). Fluorescent preparations of proliferating PC12 cells were documented at 600-fold magnification (Nikon Eclipse TE2000-U). Image acquisition of differentiated PC12 cells was performed with a Zeiss Axiophot at 1000-fold magnification.

### Cell proliferation and cell death analyses

PC12 cell proliferation upon stimulation with 50 and 100 ng/ml VEGF_165 _(R&D Systems) was assayed after 24 h, 48 h, and 72 h using the CellTiter 96^® ^AQueous One Solution Cell Proliferation Assay (Promega). These assays were performed using two different cell densities (2 × 10^4 ^cells/cm^2 ^and 6.7 × 10^4 ^cells/cm^2^) and full serum as well as serum-deprived conditions (0.1% and 0.4% horse serum). In addition, increasing VEGF_165 _concentrations from 0.2 to 400 ng/ml were added to PC12 cells cultured in 5% fetal bovine serum, and [^3^H]thymidine incorporation was measured 72 hours after onset of stimulation. The fluorometric CaspACE™ Assay System and western blot analyses with an anti-cleaved caspase-3 antibody (1:1000, Cell Signaling, #9664) were used to monitor apoptosis of differentiated PC12 cells (see below). All experiments were performed in triplicate.

### SDS-PAGE/Western blot analyses of differentiated apoptotic PC12 cell lysates

PC12 cells were grown to 50% confluence in 10 cm tissue culture dishes and NGF-differentiated for 72 h in serum-free DMEM. For induction of apoptosis, cells were washed three times with serum-free DMEM and incubated for 7.5 h under serum-deprived conditions in NGF-free DMEM. After addition of exogenous recombinant human VEGF_165 _(R&D Systems) for the indicated time frames, PC12 cells were washed once with ice-cold PBS containing 100 μM sodium orthovanadate, followed by centrifugation at 5000 rpm for 5 min at 4°C. The cells were lysed in 200 μl of ice-cold lysis-buffer (HEPES, pH 7.8, 150 mM KOAc, 50 mM ß-glycerolphosphate, 25 mM NaF, 10 mM MgCl_2_, 5 mM EGTA, 1 mM EDTA, 10% glycerol, 1% Triton X-100, 0,05% (v/v) ß-mercaptoethanol, 1 μg/ml aprotinin, 6 μg/ml chymostatin, 1 μg/ml leupeptin, 1 μg/ml pepstatin A, 1 mM PMSF, 1 mM sodium orthovanadate). Supernatants were collected after centrifugation at 14 000 rpm for 10 min at 4°C. Standardized samples containing 50 μg of whole protein (Bradford assay) were separated using 10–20% gradient gels. Proteins were wetblotted onto nitrocellulose and probed with anti-phospho-ERK1/2 (1:2000, Sigma, M8159) or anti-phospho-Akt-Ser473 (1:1000, Cell Signaling, #9271) antibodies. The blots were stripped and reprobed with antibodies detecting the respective non-phosphorylated proteins (anti-ERK1, 1:1000, Santa Cruz Biotechnology, sc-94; anti-Akt, 1:1000, Cell Signaling, #9272). Horseradish peroxidase-conjugated secondary antibodies (1:2000, Dianova) were visualized by enhanced chemiluminescence detection (Western Lightning™ Chemiluminescence Reagent System, PerkinElmer). Experiments were performed in triplicate.

## Results

### Differential cell binding of VEGF and endostatins

**Figure 1:**
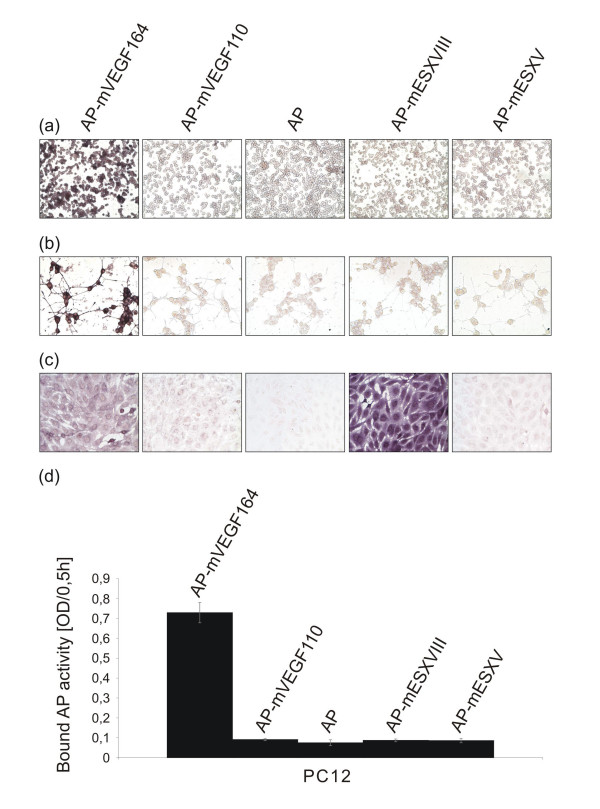
Alkaline phosphatase (AP) staining of (**a, d**) proliferating and (**b**) differentiated PC12 cells revealed binding of the AP-mVEGF_164 _affinity probe while AP-murine endostatin (AP-mESXVIII) only stained (**c**) endothelial cells.

To determine the expression profile of binding partners for murine VEGF_164_, endostatin and the collagen XV endostatin homologue on neuronal and endothelial cell lines, PC12 and CPAE cells were incubated with AP fusion proteins. The AP-mVEGF_164 _affinity probe strongly stained proliferating and differentiated PC12 cells (Fig. 1a, b). In contrast, AP-mVEGF_110 _which lacks the C-terminal heparan sulfate binding domain, the endostatin domain of collagen XVIII (AP-mESXVIII), and the collagen XV endostatin homologue (AP-mESXV) did not bind to PC12 cells. Both AP-mVEGF_164 _and AP-mESXVIII labelled CPAE cells while AP-mVEGF_110_, AP-mESXV and control AP did not (Fig. 1c). Quantitative measurement of AP fusion protein binding to proliferating PC12 cells confirmed the above results (Fig. 1d). In agreement with lack of AP-mESXVIII and AP-mESXV binding to PC12 cells, recombinant human endostatins [22] did neither promote nor inhibit PC12 cell differentiation (data not shown). Thus, PC12 cells are not a useful model for understanding endostatin effects on cells.

### PC12 cells express high-affinity VEGF receptors

**Figure 2:**
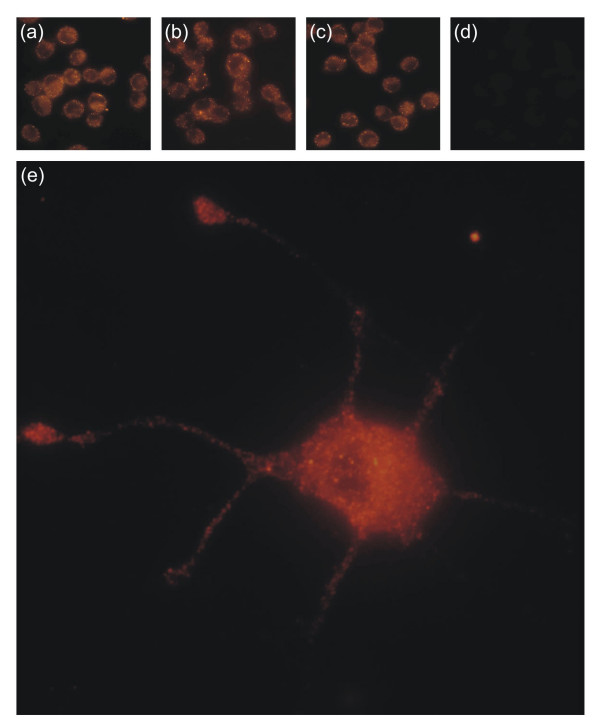
Immunodetection of VEGF receptors expressed on the cell surface of (**a-d**) proliferating and (**e**) differentiated PC12 cells. PC12 cells were stained with polyclonal antibodies against (**a**) VEGFR-1, (**b, e**) VEGFR-2, and (**c**) neuropilin-1. The primary antibody was omitted in (**d**) controls. Differentiated PC12 cells were also immunoreactive for VEGFR-1 and neuropilin-1 (data not shown).

Consistent with AP staining, it was shown by immunofluorescence that PC12 cells express the high-affinity receptor tyrosine kinases VEGF receptor 1 and 2 (VEGFR-1, VEGFR-2) as well as the low-affinity receptor neuropilin-1 (Fig. 2). These receptors are expressed on the cell surface of proliferating (Fig. 2a–c) and differentiated (Fig. 2e) PC12 cells and seem to possess a clustered morphology reminiscent of activated tyrosine kinases. Comparable results were obtained with antibodies from Santa Cruz Biotechnology and Dianova (data not shown).

### VEGF induces transient activation of ERK1/2 and Akt kinase in differentiated apoptotic PC12 cells

**Figure 3:**
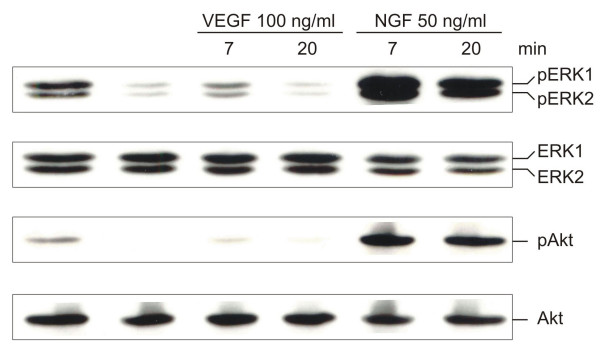
Western blot analyses of VEGF-induced signal transduction in differentiated PC12 cells after NGF withdrawal. Lysates of control cells maintained in the presence of NGF were loaded in lane 1. NGF-deprived PC12 cells (lane 2) treated with VEGF_165 _(lanes 3, 4) or NGF (lanes 5, 6) demonstrated that VEGF_165 _induced transient activation of ERK1/2 and Akt after 7 min. In contrast, NGF produced a stronger and persistent phosphorylation of ERK1/2 and Akt than VEGF_165_.

While the addition of VEGF_165 _had no effect on PC12 cell proliferation and neurite formation, a consistent but non-significant reduction of caspase-3 activity became apparent when VEGF_165 _was administered to differentiated apoptotic PC12 cells (data not shown). To analyze VEGF signaling in PC12 cell survival, the activation of extracellular signal-regulated kinases ERK1/2 and Akt was examined by Western blot analyses using phospho-specific antibodies and their respective non phosphorylated counterparts. Control cultures demonstrated that ERK1/2 (p44/p42 MAPK) and Akt activation are sustained for 79.5 h in the presence of NGF (Fig. 3, lane 1). Removal of NGF for 7.5 h after 72 hours of differentiation led to a significant reduction of ERK1/2 and Akt phosphorylation (Fig. 3, lane 2). Exogenous addition of 100 ng/ml VEGF_165 _to NGF-deprived PC12 cells resulted in transient ERK1/2 activity within 7–10 min which decreased almost to control levels 20 min after stimulation (Fig. 3, lanes 3, 4, and data not shown). Higher concentrations of recombinant VEGF (200 ng/ml) did not increase or prolong ERK1/2 activity (data not shown). Similar activation kinetics were observed for phosphorylation of Akt at serine 473 (Fig. 3). For comparison of signaling mechanisms, apoptotic PC12 cells were also NGF-stimulated. NGF-stimulation induced a much more pronounced and sustained activation of ERK1/2 and Akt which was even more prominent than in NGF treated non-apoptotic control cells (Fig. 3, lanes 5, 6).

## Discussion

We here report that endostatin affinity probes derived from collagens XVIII and XV do not bind to PC12 cells indicating that these cells do not express endostatin cell membrane receptors. This observation is consistent with absent effects of recombinant endostatins on neurite outgrowth (data not shown) and lack of binding of AP-mESXVIII and AP-mESXV to murine embryonal nerve tissues [19]. AP-mESXVIII predominantly labelled blood vessels while AP-mESXV binding was restricted to the lense capsule [19] which correlates with the current results that only AP-mESXVIII, but not AP-mESXV, strongly stained CPAE cells. As opposed to the endostatin affinity probes, AP-mVEGF_164 _showed strong binding to PC12 cells. Undifferentiated PC12 cells were known to express VEGF to stimulate angiogenesis [23]. We now demonstrate that proliferating and differentiated PC12 cells also express VEGFR-1 and -2 and NRP1. NRP1 acts as an isoform-specific VEGF co-receptor which only binds VEGF_165 _[24]. Since C-terminally deleted AP-mVEGF_110 _did not bind to PC12 cells, NRP1 appears to be required for the interaction of VEGF_165 _with PC12 cells.

Despite prominent expression of VEGF receptors on PC12 cells, exogenous VEGF_165 _had no effect on PC12 cell proliferation and neurite formation. One reason might be endogenous VEGF-expression of proliferating PC12 cells which is downregulated only 48 h after induction of differentiation with NGF [23]. This would also explain the slight anti-apoptotic effect of VEGF_165 _on PC12 cells that had been differentiated for three days prior to VEGF_165 _stimulation. However, only an insignificant decrease of cell proliferation could be observed upon treatment with an antibody against rat VEGF_164 _(data not shown). Thus, our data are in line with the observation that VEGFR-1-expressing cells show a poor mitogenic response to VEGF stimulation [5]. Analysis of VEGF_165_-induced signal transduction in differentiated apoptotic PC12 cells demonstrated activation of ERK1/2 and Akt. The transient nature of VEGF_165_-triggered ERK1/2 phosphorylation in PC12 cells provides a further explanation for the observation that VEGF_165 _was not able to induce PC12 cell differentiation which is known to require sustained activation of the MAPK cascade [25]. The inefficient rescue of PC12 cells from apoptosis through VEGF_165 _is likely also due to its short-lived and relatively small effect on ERK1/2 and Akt.

VEGF-induced neuroprotective signaling via VEGFR-2, NRP1 and the two above-mentioned signaling cascades was shown in hypoxic and glucose-deprived hippocampal neuron × neuroblastoma (HN33) hybrid cells [11], in rat primary hippocampal neurons that had been exposed to glutamate [12], and in hypoxic murine primary cortical neurons [13]. In these in vitro model systems of cerebral ischemia, no comparison of VEGF and NGF activation kinetics was performed. Our results on growth factor stimulated PC12 cells show that both the anti-apoptotic effect and the activation of ERK1/2 and Akt were transient and minor when compared to NGF. It remains to be clarified whether this is a consequence of experimental conditions, cell line-specific or a general feature of VEGF-induced neuroprotection.

## Conclusion

Based on experiments using growth factor deprivation, the present study suggests that NGF protects neuronal cells from cell death much more efficiently than VEGF_165_. The significant NGF-induced reduction of caspase-3 activity in differentiated apoptotic PC12 cells correlates with a much more pronounced and prolonged activation of downstream effectors when compared to VEGF_165_. Thus, the angiogenic compound VEGF_165 _may only be a minor player in neurogenesis and neuronal survival and may only have little therapeutic and side effects on neuronal cells.
